# Multi-modal novelty and familiarity detection

**DOI:** 10.1186/1471-2202-14-S1-P65

**Published:** 2013-07-08

**Authors:** Christo Panchev

**Affiliations:** 1 Department of Computing, Engineering and Technology, University of Sunderland, Sunderland SR6 0RD, UK

## 

Presented is further development of the architecture presented in [1] where a top-down feature-based and spatial attention have been incorporated in a large scale visual module and novelty and familiarity detectors based on the model presented in [2]. These have been developed in the perceptual (visual and auditory) and motor modalities. In addition to the novelty/familiarity detection shown in [2,3], the architecture is able to partially recognise familiar features in each perceptual modality, and furthermore in a distributed fashion activate associated familiar features from one perceptual modality to another and/or to the motor programmes and affordances. The architecture is implemented on a mobile robot operating in a dynamic environment. The proposed distributed multi-modal familiarity detection integrated in the architecture improves the recognition and action performance in a noisy environment, as well as contributing to the multi-modal association and learning of novel objects and actions.

**Figure 1:**
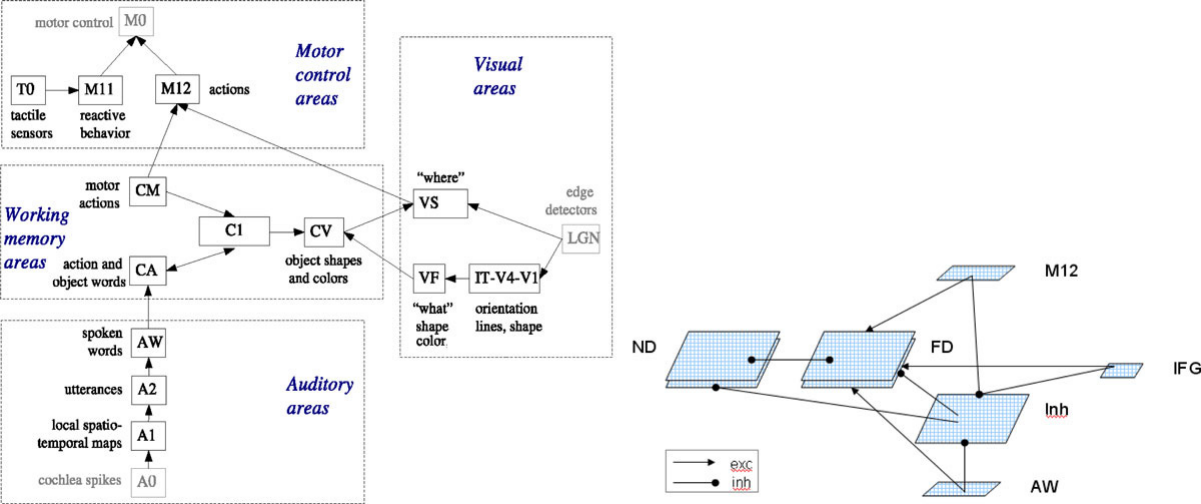
**Multi-modal Robot Control Architecture (left)**. Novelty and familiarity Detectors (right).

The neural network is built on integrate-and-fire spiking neurons with active dendrites (ADDS) and trained with STDP rule presented in [4]. The overall architecture is shown in Figure 1 (left). It integrates representation of concepts in different modalities. The distributed representation in the working memory area achieve temporal binding via phase-locking, whereas different concepts are separated in time. The robot implementation provides interpretation and execution of simple instructions given via the auditory area and interpreted in the working memory which is integrating sequential language input into instruction representation. The separate entities of the instruction and the instruction as a whole run in nested gamma-theta oscillations. The novelty and familiarity detectors network is shown on Figure 1 (right). The familiarity detector (FD) and novelty detector (ND) areas are 25x25 ADDS neurons trained with STDP. After training approximately 68% (± 15 in the different trials / training sessions) of the FD neurons become unimodal action/object specific. About 22% (±15) of the ND neurons were also observed to become unimodal action/object specific, responding to when a particular object/action are not present in the verbal instruction or the visual field.
